# Texture Analysis in Uterine Cervix Carcinoma: Primary Tumour and Lymph Node Assessment

**DOI:** 10.3390/diagnostics13030442

**Published:** 2023-01-26

**Authors:** Paul-Andrei Ștefan, Adrian Coțe, Csaba Csutak, Roxana-Adelina Lupean, Andrei Lebovici, Carmen Mihaela Mihu, Lavinia Manuela Lenghel, Marius Emil Pușcas, Andrei Roman, Diana Feier

**Affiliations:** 1 Department of Biomedical Imaging and Image-Guided Therapy, General Hospital of Vienna (AKH), Medical University of Vienna, 1090 Vienna, Austria; 2 Anatomy and Embryology, Morphological Sciences Department, “Iuliu Haţieganu” University of Medicine and Pharmacy, Victor Babeș Street, Number 8, 400012 Cluj-Napoca, Romania; 3 Radiology and Imaging Department, County Emergency Hospital, Clinicilor Street, Number 3–5, 400006 Cluj-Napoca, Romania; 4 Clinical Surgery Department 1, Emergency Clinical County Hospital Oradea, 65 Gheorghe Doja Street, Bihor, 410169 Oradea, Romania; 5 Radiology and Imaging, Surgical Specialties Department, “Iuliu Haţieganu” University of Medicine and Pharmacy, Clinicilor Street, Number 3–5, 400006 Cluj-Napoca, Romania; 6 Histology, Morphological Sciences Department, “Iuliu Hațieganu” University of Medicine and Pharmacy, 400349 Cluj-Napoca, Romania; 7 Obstetrics and Gynecology Clinic II, County Emergency Hospital Cluj-Napoca, 21 Decembrie 1989 Boulevard, Number 55, 400094 Cluj-Napoca, Romania; 8 Oncological Surgery and Gynaecologic Oncology, Surgery Department, “Iuliu Hatieganu” University of Medicine and Pharmacy, 400006 Cluj-Napoca, Romania; 9 General Surgery Department, Institute of Oncology “Prof. Dr. Ion Chiricuta”, 400006 Cluj-Napoca, Romania; 10 Radiology and Imaging Department, Institute of Oncology “Prof. Dr. Ion Chiricuta”, 400006 Cluj-Napoca, Romania

**Keywords:** cervical cancer, computer-aided diagnosis, gynecological malignancy, magnetic resonance imaging, MRI, radiomics, texture analysis

## Abstract

The conventional magnetic resonance imaging (MRI) evaluation and staging of cervical cancer encounters several pitfalls, partially due to subjective evaluations of medical images. Fifty-six patients with histologically proven cervical malignancies (squamous cell carcinomas, *n* = 42; adenocarcinomas, *n* = 14) who underwent pre-treatment MRI examinations were retrospectively included. The lymph node status (non-metastatic lymph nodes, *n* = 39; metastatic lymph nodes, *n* = 17) was assessed using pathological and imaging findings. The texture analysis of primary tumours and lymph nodes was performed on T2-weighted images. Texture parameters with the highest ability to discriminate between the two histological types of primary tumours and metastatic and non-metastatic lymph nodes were selected based on Fisher coefficients (cut-off value > 3). The parameters’ discriminative ability was tested using an k nearest neighbour (KNN) classifier, and by comparing their absolute values through an univariate and receiver operating characteristic analysis. Results: The KNN classified metastatic and non-metastatic lymph nodes with 93.75% accuracy. Ten entropy variations were able to identify metastatic lymph nodes (sensitivity: 79.17–88%; specificity: 93.48–97.83%). No parameters exceeded the cut-off value when differentiating between histopathological entities. In conclusion, texture analysis can offer a superior non-invasive characterization of lymph node status, which can improve the staging accuracy of cervical cancers.

## 1. Introduction

Cervical cancer is the third most common gynaecological malignancy worldwide with an estimated incidence of more than 500,000 new cases and more than 250,000 deaths per year [1]. These tumours continue to be the fourth most prevalent malignancy and the fourth most common cause of cancer death in women despite advances in their identification and treatment. [2]. There is an obvious discrepancy in the diagnosis, management, and survival between developed and developing countries [3]. In the latter, these differences are partially caused by limited access to healthcare services, including advanced imaging techniques.

There are two main types of uterine cervix carcinomas. Squamous cell carcinomas (SQCs) are the most common histological entity, while adenocarcinomas (ADKs) account for 10–25% of all cases, with a gradually increasing incidence in recent years [4]. Most studies consider ADKs as having a worse prognosis [5,6], while other research did not detect significantly different outcomes of squamous and non-squamous carcinoma in early cancer stages [7]. Therefore, the histologic impact on tumoral prognosis is still in debate.

According to the International Federation of Gynecology and Obstetrics (FIGO) criteria, adopted in 1958 and revised in 2018, cervical cancer is currently the only gynaecological malignancy that is still staged clinically. [8]. The latter introduces cross-sectional imaging in the staging of these tumours, which brings two major benefits: the assessment of retroperitoneal lymph nodes with the inclusion of a new stage (IIIC), and the 2-cm cut-off dimension of primary tumours used for fertility-sparing trachelectomy [8]. However, it is not stipulated which imaging technique is best suited for assessing cervical tumours and lymph node status.

The radiological characterization of cervical cancers has a great impact on their subsequent management. The classical imaging modalities used for the morphological characterization of these malignancies include ultrasonography, computer tomography (CT), and magnetic resonance imaging (MRI) [9]. Additionally, relatively newly developed imaging techniques, such as positron emission tomography-computed tomography (PET-CT), diffusion-weighted imaging (DWI) with the measurement of the apparent diffusion coefficient (ADC), and dynamic contrast-enhanced imaging (DCE) via perfusion parameters, have the potential to improve the diagnosis of these tumours [10,11,12].

Despite the rapid development of these techniques, there are still several pitfalls in assessing parametrial invasion and adenopathy detection. The sensitivity of imaging modalities in the diagnosis of parametrial invasion can range between 53–74% for MRI and 42–55% for CT [9,13]. However, the lymph node status is more difficult to establish. Lymph node metastases (LNM) can be detected with a 29% to 86% sensitivity by common imaging techniques, mostly because micro-metastatic lymph nodes with a normal size are not detected through the macroscopic evaluation of medical images [14,15]. It is crucial to improve these issues, since the oncological treatment depends on the tumoral staging, which is modified by the detection of parametrial and lymph node involvement [16].

It is desirable that certain histopathological features of cervical cancers could also be reflected in medical images, but they are impossible to be quantified by the naked eye. As a novel technique to extract quantitative data (texture parameters) from almost all types of imaging examinations, texture analysis (TA), has recently gained popularity in the imaging research field. TA appraises pixel intensity and distribution patterns, offering an objective description of image content [17]. Previous research evaluating the utility of TA applied to MRI images of cervical cancers yielded promising results. Becker and colleagues [18] showed that texture parameters extracted from DWI images can predict tumour differentiation and nodal status; another study conducted by Meng et al. [19] showed that parameters computed from T2 sequences and ADC maps could predict cervical cancer’s recurrence, and Sun and colleagues [20] concluded that TA parameters extracted from T1- and T2-weighted images can be used as pretreatment predictors of chemotherapy response.

The objective of this study was to assess the usefulness of TA applied to T2-weighted images of cervical cancers in the pre-treatment stage. Our goal was to determine whether texture criteria may help discriminate between FIGO stages, identify the histological tumour type, and assess the lymph node status.

## 2. Materials and Methods

### 2.1. Study Group

This single-institution, retrospective pilot study has been approved by the institutional review board (Ethical Committee of the “Iuliu Hațieganu” University of Medicine and Pharmacy Cluj-Napoca; No 50/11.03.2019). Written informed consent was provided by all patients.

A keyword search in our institution’s database was conducted in order to identify all patients with cervical cancer who underwent abdominopelvic MRI examinations before surgical or radio-chemotherapy treatment between November 2018 and May 2020, using the words: “cervical + cancer/+ tumor/+ mass” and “cervix + cancer/+ tumor/+ mass”. The original search showed 271 reports, which were inspected by one researcher. All the cases without uterine cervical cancer were excluded (*n* = 32). The remaining 229 participants’ medical records were collected from our healthcare unit’s archive and examined for information on illnesses. The following exclusion criteria were applied: subjects who did not undergo colposcopy with biopsy in our healthcare unit (*n* = 29), those with a lack of initial FIGO staging (*n* = 3), patients who did not undergo oncological or surgical treatment and follow-up after the initial MRI examination (*n* = 43), those who received follow-up examinations after surgery or chemoradiation therapy (*n* = 97), and those for whom imaging artifacts affected the T2-weighted sequences (*n* = 11).

### 2.2. Imaging Protocol

Abdominopelvic MRI acquisitions were performed with a 1.5 T scanner (General Electric Sigma Excite^®^, GE Medical Systems, Milwaukee WI, USA). The administration of 20 mg hyoscine butyl bromide (Buscopan^®^, Sanof SA, Paris, France) was used to diminish intestinal peristalsis. For the pelvic protocol, a phased array coil was used, and the following sequences were performed: axial T1 fast spin-echo (FSE), sagittal T2 fast recovery fast spin-echo (FRFSE), oblique axial T2 FRFSE, oblique axial T2 FRFSE, axial T2 FRFSE, axial diffusion-weighted sequences with three b values (0, 800, 1000 s/mm^2^). For abdominal examination, a phased array coil was used, and the following sequences were performed with breath-hold: axial and coronal two-dimension (2D) fast imaging employing steady-state acquisition (FIESTA), axial T2, axial T1 fast spoiled gradient echo (FSPGR) with fat saturation (FatSat). Subsequently, 18–20 s following intravenous injection of gadolinium dimeglumine (Magnevist^®^, Berlex, Wayne, NJ, USA) 0.1 mol/L per kg body weight, a three-dimensional (3D) liver acquisition with volume acceleration (LAVA) and a post-contrast axial T1 FSPGR FatSat were obtained. Pelvic post-contrast imaging was acquired 5 min after intravenous injection with axial T1 FSE. Each MRI acquisition included an oblique axial T2 FRFSE (echo time (TE), 102; repetition time (TR), 3350; field of view (FOV), 26 cm, section thickness, 5 mm; spacing, 0 mm; and number of excitations (NEX), 3) and a sagittal T2 FRFSE sequences (TE, 85; TR, 3100; FOV, 24 cm; section thickness, 5 mm; spacing, 0.5 mm; and NEX, 4), which were the only ones further processed in the current study. The oblique axial T2 FRFSE and the sagittal T2 FRFSE were the only sequences used for the texture analysis.

### 2.3. Texture Analysis Protocol

The radiomic method entails four stages: image segmentation utilizing a region of interest, feature extraction, feature selection, and prediction. Two radiologists (with a combined expertise of 10 years in pelvic genital oncology MRI) who were aware of the final diagnosis assessed all of the exams on a dedicated workstation.

MRI imaging features and pathological analysis were considered when assessing the lymph node status. A maximum lymph node diameter of less than 1 cm and no morphological tumour changes were considered as advocating for benignancy. LNM was considered when at least two of the following criteria were met: a short lymph node axis of >1 cm, the presence morphological changes advocating for malignancy (necrosis, invasive contour, advanced architectural disorganization), or when indicated by the pathological analysis following surgical dissection. On the sagittal T2-weighted images (T2WI), the same radiologists correlated the lymph nodes’ imaging appearance with the available medical data and selected and marked only one lymph node considered representative from every patient. Tumour evaluation was performed on the oblique axial T2WI. The examinations were then anonymized, and the two T2-weighted sequences were retrieved in DICOM format (Digital Imaging and Communications in Medicine).

**Figure 1:**
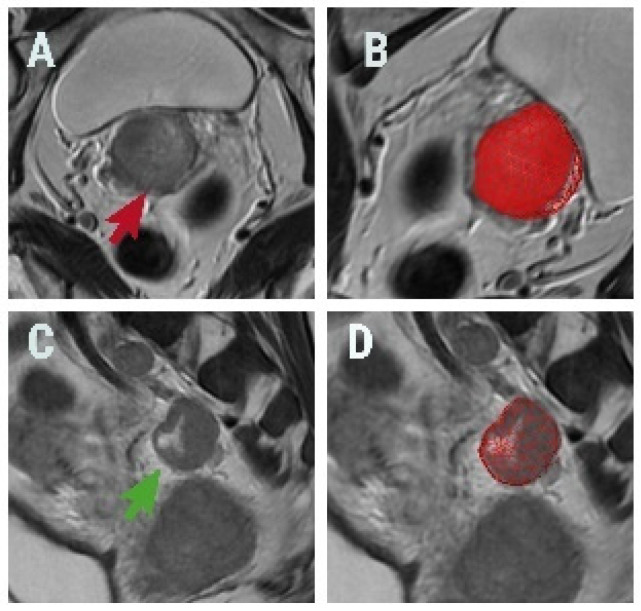
(**A**) Oblique axial T2-weighted image of a 55-year-old patient with squamous cervical carcinoma (red arrow) and (**B**) a three-dimensional (3D) region of interest (ROI) (red) covering the tumour. (**C**) Sagittal T2-weighted image of a 61-year-old patient with histologically proven metastatic lymph node (green arrow) and (**D**) the 3D ROI (red) covering the lesion.

A third radiologist, blinded to the clinical outcome, imported the sequences in a freely available texture analysis software, MaZda version 5 [21]. Each primary tumour and selected lymph node were separately segmented using a 3D region of interest (ROI) which crossed all the slices in the sequence where the targeted lesions were observed. For the definition of each ROI, a semi-automatic level-set technique was used supervised by the same radiologist who also made the necessary adjustments (Figure 1). Texture analysis’ results are influenced by inter- and intrascanner effects [22,23]. To diminish these effects, the ROI contents were normalized between the mean and three standard deviations.

Over 300 parameters were extracted from the texture analysis of each ROI. These parameters, originating from the texture analysis of each ROI, yielded more than 300 parameters. These parameters were calculate using the absolute gradient, the run-length matrix, the co-occurrence matrix (GLCM), wavelet transformation, and histogram analysis. A reduction technique was applied to narrow the number of parameters, since such large data cannot be statistically analysed. In order to find the most relevant parameters to differentiate between groups, the Fisher selection method was applied. This method provided the selection of ten texture features that have a high discriminatory potential [24]. Fisher coefficients (F, the ratio of between-class to within-class variance) which had a value of at least 3 were considered as having good discriminatory potential.

Regarding the parameter name generated by the MaZda software, it represents an abbreviation of the particular feature produced by the extraction algorithm. The first letter designates the colour channel, whereas the letter “C” indicates that a black-and-white image was computed. The next letters (H, V, Z, and N) symbolize the four directions between the pixels taken into account (horizontal, vertical, 45°, and 135°, respectively). The letter “S” symbolizes a μ ± 3σ (μ = grey-level mean; and σ = grey-level standard deviation) method used for the normalization of ROIs. The number following the direction code defines the distance in the GLCM algorithm between the pixels being taken into account [25].

### 2.4. Image Classification and Statistical Analysis

Comparisons of texture parameters were performed to distinguish between the two histopathological entities of cervical carcinoma (SQCs and ADKs) and between non-metastatic and metastatic lymph nodes. Two methods were used to assess the discriminatory power of the texture features.

Firstly, the set of ten parameters selected by the Fisher method was further imported into the B11 program, part of the MaZda package, which allowed us to further investigate the utility of selected texture features to discriminate between selected groups by the use of classifiers. A classifier based on k nearest neighbour (KNN) was used to test the ability of texture parameters to distinguish between selected groups. The KNN test’s performance was quantified through its accuracy (expressed as a percentage of correctly classified lesions/total).

Secondly, the Mann–Whitney U test was used to compare the absolute values of texture parameters which were greater than the Fisher coefficients’ cut-off value. The parameters having *p* values less than 0.05 on the univariate analysis were subjected to receiver operating characteristic (ROC) analysis, and the area under the curve (AUC) was determined with 95% confidence intervals (CIs). Independent predictor factors for the diagnosis of LNM were found using multiple regression analysis using a “enter” input model, and the coefficient of determination (R-squared) was calculated. ROC analysis was used to assess the predictors’ diagnostic value. A dedicated piece of software called MedCalc version 14.8.1, which is available for purchase, was used for statistical analysis (MedCalc Software, Mariakerke, Belgium).

## 3. Results

Following application of the inclusion and exclusion criteria, 56 of the 271 patients referred to our department throughout the study period were included in the analysis. (mean age, 51.93 years; age range, 28–77 years; standard deviation, ±12.43 years). Histopathological diagnosis after biopsy identified squamous carcinoma in 42 subjects and adenocarcinoma in 14 patients. The clinical staging was assessed using the 2018 FIGO classification: IB (in 7 patients), IIA (in 8 patients), IIB (in 24 patients), IIIB (in 3 patients), and stage IIIC (in 13 subjects).

Imaging and pathology results were referenced when assessing the lymph node status. Thirty-nine patients were considered as having no tumoral lymph node involvement, as having a short lymph node axial diameter of less than 1 cm and no morphological changes advocating for malignancy. The short axis of all considered LNMs exceeded 1 cm. Surgical removal followed by pathological confirmation of lymph node tumoral involvement was performed for 12 patients. Morphological changes advocating for malignancy were represented by (necrosis, *n* = 1; irregular and infiltrative contour, *n* = 1; advanced architectural disorganization, *n* = 3). No paraaortic lymph node met the inclusion criteria. The texture parameters with the highest discriminative potential selected by the Fisher method for every comparison are shown in Table 1.

The comparison between squamous cell carcinomas and adenocarcinomas showed that no texture parameter exceeded the Fisher coefficient cut-off value. The KNN system misclassified all data vectors from ADKs. The highest-rated texture feature (F = 0.65) in the comparison between the two histological types of malignancy was the inverse difference moment (CZ5S6InvDfMom). The median values of this parameter were higher for SQCs (median value, 16.5; interquartile range (IQR), 0.11–210.13) than for ADKs (median value, 0.11; IQR, 0.05–0.16).

**Figure 2:**
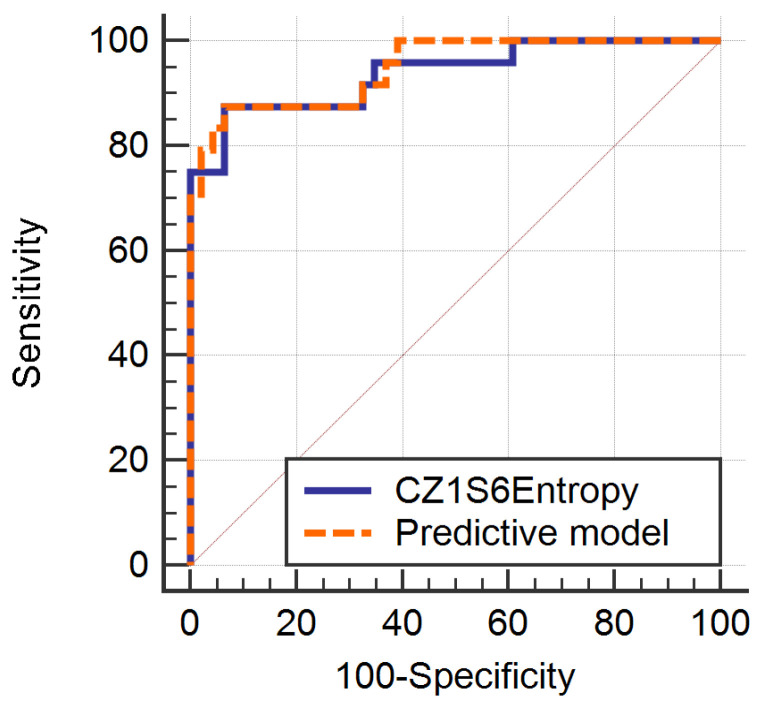
Receiver operating characteristic (ROC) curve for the multivariate analysis in metastatic lymph node detection compared to CZ1S6Entropy.

**Figure 3:**
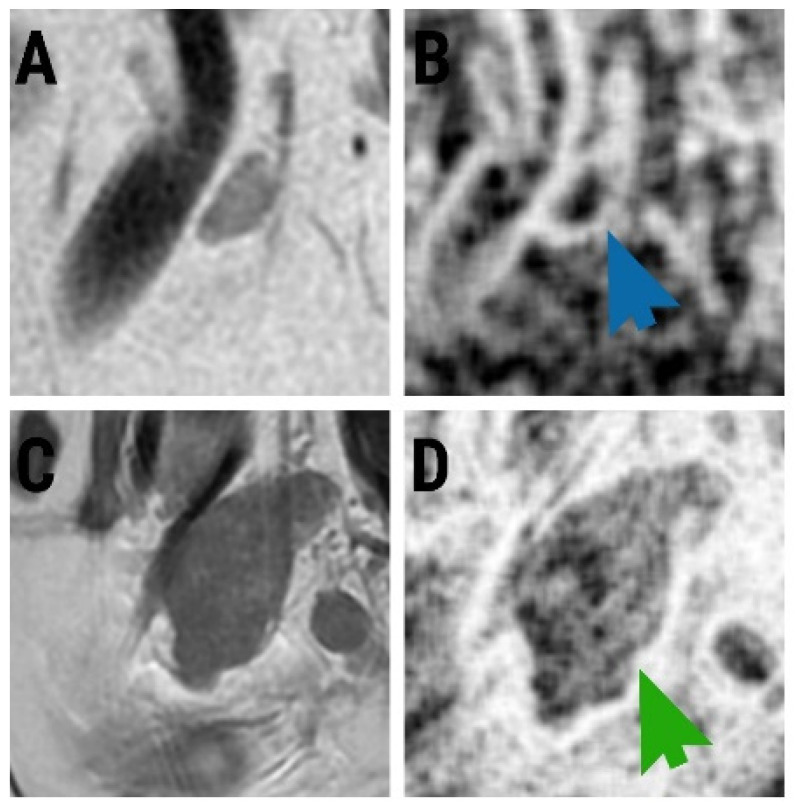
(**A**) A sagittal T2-weighted image of a non-tumoral lymph node in a 58-year-old patient; (**B**) generated map of this image based on CZ1S6Entropy parameter (blue arrow pointing to the lesion). (**C**) A sagittal T2-weighted image of a 61-year-old patient with pathologically confirmed metastatic lymph node; (**D**) generated map of this image based on CZ1S6Entropy parameter (green arrow pointing to the lesion).

The median lymph node diameter was 7.31 mm (IQR, 3.2–9.64 mm) for benign (non-metastatic) and 17.83 mm (IQR, 12.7–20.6 mm) for malignant (LNM) nodes. When differentiating metastatic from non-metastatic lymph nodes, all parameters from the Fisher-selected set exceeded the cut-off value. The entire feature set consisted of computation variations of the entropy parameter. The ANN misclassified 6.25% (accuracy, 93.75%) of the data vectors, all consisting of images of metastatic nodes that were considered non-malignant. The comparison between the two lymph node statuses, based on the absolute values of each parameter, was statistically significant (*p* < 0.0001) (Table 2). The individual ROC analysis showed adequate discriminatory potential for each entropy variation (Table 3). In multivariate analysis, according to multiple regression, only one parameter (CZ1S6Entropy) was an independent predictor of metastatic lymph node (R2 = 0.587) (Table 4). This was also the parameter that held the highest Fisher coefficient (F = 6.25). Considered together, the ten variations of the entropy parameter were able to predict metastatic lymph nodes with 87.5% (67.6–97.3%) sensitivity and 93.48% (82.1–98.6%) specificity. The ROC curve comparison between CZ1S6Entropy and the multivariate analysis did not yield statistically significant results (*p* = 0.427) (Figure 2). Figure 3 displays visual maps generated on the basis of texture parameters.

## 4. Discussion

Squamous cell carcinomas and adenocarcinomas are the most common histopathological types of uterine cervical cancers [26]. Because classic imaging diagnostic techniques are not able to differentiate the two histological tumour types, a colposcopy with biopsy remains mandatory [7]. However, oncological management does not significantly differ for the two types, and therefore the histopathological appurtenance can be considered an independent prognostic factor [27].

This study failed to differentiate between the two types of cervical primary tumours based on texture features. The KNN classifier did not recognize any ADKs as being a separate histological entity from SQCs. However, ADKs measured higher values of the InvDfMom parameter compared to SQCs. This parameter measures the degree of homogeneity within an image. Its values rise when more pixel pairs are close to the grey-scale value, resulting in higher values for homogeneous images [28]. This fact is probably a consequence of the unequal distribution of groups. Since the SQC group comprised almost three times more cases than the ADK, it is possible that some of the lesions comprising the first group had more heterogeneous differentiation degrees, or the MRI appearance was contaminated by necrosis or bleeding. A similar study conducted by Becker et al. [18] also observed no correlation between texture parameters extracted from the ADC maps and the histological subtype of cervical carcinomas. On the contrary, the research of Ciolina et al. [29], which also extracted texture features from T2WI but used different software (TexRad) for analysing the data, concluded that ADKs and SQCs can be well differentiated by the parameters mean and skewness (*p* = 0.002). These good results may be due to the use of more advanced software used for image processing. TexRad allows the appliance of multiple anatomical filters that enhance intensity-variation features of different sizes corresponding to the spatial scale of the filter itself [29]. It is possible that the application of these filters would result in parameters that better analyse the intrinsic properties of the examined tissues.

Lymph node involvement in cervical cancer is an important prognostic factor, requiring a radical change in treatment [30]. Even following specific therapy, patients with LNM show a reduction in the overall 5-year survival rate [31]. Additionally, Meng J et al. [19] demonstrated that tumour recurrence is significantly higher in patients with positive lymph nodes, compared to the negative group. In addition, LNM has a similar incidence in early (10–30%) compared to locally advanced cervical cancer (15–30%) [32,33].

There are a few pitfalls in the conventional MRI evaluation of LNM in cervical cancer. By simple qualitative assessment, LNM could be diagnosticated with high specificity (91%) but low sensitivity (57%) in a study conducted by Liu B et al. [34]. Size measurements can predict LNM in cervical cancer with a high specificity (96.8–97.9%) but again low sensitivity (43.5–62.2%) [35,36]. More advanced MRI techniques, such as DWI via the ADC values, were proven to be more accurate in identifying LNM (83.3% specificity, 74.7% sensitivity, and 78.4% accuracy) [37]. However, later research [37] included only lymph nodes that exceeded 5 mm in diameter, most likely because of the ADC maps’ low resolution. Nonetheless, the gold standard for lymph node diagnosis remains surgery followed by pathological analysis. However, unnecessary lymphadenectomy may lead to post-surgical morbidities, such as infection or nerve or vessel lesions [38]. Therefore, it is beneficial to detect the presence of lymph node metastases before treatment using non-invasive imaging techniques.

Our results showed that the entire feature set, composed of ten computation variants of entropy, was able to distinguish metastatic from non-metastatic lymph nodes. One single variation, CZ1S6Entropy (entropy computed from a grey-scaled image, using a 45° direction code and an inter-pixel distance of 6, from an ROI normalization of μ ± 3σ), proved to be an independent predictor for LNM. However, CZ1S6Entropy diagnostic performance was not statistically different from the overall multivariate model, most likely because the parameters were highly-corelated with each other. Entropy parameters measure the disorder of pixel intensity within an image. The values increase when there is a non-uniform image content, and decrease otherwise [39,40,41]. All ten entropy variations held higher values for LNMs than for benign nodes, probably because the tumoral cells produced high disorder in the normal lymph node architecture. This observation can also be supported by the two entities’ appearance on the maps generated based on the CZ1S6Entropy parameter

Moreover, another classifier used for the same scope (based on artificial neural networks) showed better accuracy for LNM classification than the previously documented ADC values [37] (93.75% versus 78.4%). Overall, texture parameters individual and combinate sensibility for LNM diagnosis exceeded the ones previously reported for qualities, size, and ADC evaluations [34,35,36,37]. The individual specificity recorded by texture parameters, however, was almost equal with the one found for conventional imaging assessments [34,35,36,37], and even exceeded it in a few isolated cases. These promising results indicate that TA can augment the tumoral lymph node diagnosis and probably offer a non-invasive alternative to the present surgical gold standard. The study conducted by Becker et al. [18] also observed that texture analysis results were able to differentiate between metastatic and non-metastatic lymph nodes in cervical cancer. However, the workflow was almost entirely different in this study [18]: TA was performed on a single slice on ADC maps using MATLAB software (v2016b, The MathWorks Inc., Natick, MA, USA); the lymph node status was histologically confirmed for all 23 subjects, and two parameters derived from the histogram analysis (skewness, *p* = 0.04; kurtosis, *p* = 0.02) were statistically significant when comparing the two groups. However, the histogram parameters only reflect the pixel intensity characteristics, and not the actual spatial relation of pixel intensities, for which reason they are often referred to as non-textural attributes [18,25,42,43,44].

In summary, our findings suggest that the whole lesion texture analysis of primary cervical cancer and lymph nodes can successfully differentiate metastatic from non-metastatic, but failed to distinguish between the two histopathological entities of primary tumours. The presence of metastatic lymphadenopathy is one of the most important predictive factors for the treatment and evolution of cervical cancer [34,37]. Thus, TA can offer a non-invasive quantitative appreciation of lymph node status that also comes at a low cost, and is suitable for access in developing countries (or smaller medical centres without modern radiology departments), since only the radiomics software and the basic examination sequences are needed. It is possible that our results regarding lymph node tumoral involvement could be successfully applied to other pathologies. Additionally, because we used examinations provided by the same MRI machine and following the same protocol, we created an adequate classification environment for the KNN.

The present study had several limitations. Firstly, the cohort was rather small: 56 patients were included, with 14 cases of ADKs. This, however, is in accordance with the overall incidence of this histopathological entity [26]. Secondly, some of the cases were advanced cervical cancers that were managed conservatively, so we also categorized metastatic lymph nodes based on their imaging appearance. Thirdly, due to the retrospective nature of the study, there may have been selection bias. In this regard, the study requires validation by larger prospective research.

## 5. Conclusions

Clinical examination has supreme importance in the evaluation of cervical tumours. Imaging is required for a detailed characterization of loco-regional extension, but the classic imaging approaches are insufficient for lymph node assessment, which is one of the most important prognostic factors in cervical cancer. Texture analysis can offer a superior non-invasive characterization of lymph node status, which can improve the staging accuracy of cervical cancers.

## Figures and Tables

**Table 1:** Texture parameters selected by the Fisher method as having the highest discriminatory potential between compared groups. Parameters with adequate discriminatory potential (F > 3) are marked in bold.

Compared Groups	Texture Parameter Sets
Squamous cell carcinoma and adenocarcinoma	CZ5S6InvDfMom (F = 0.65), Teta4 (F = 0.65), Teta3 (F = 0.65), CN3S6InvDfMom (F = 0.63), CV3S6Contrast (F = 0.58), CV3S6Correlat (F = 0.58), CZ2S6DifVarnc (F = 0.57), CV4S6Contrast (F = 0.57), CV4S6Correlat (F = 0.55), CZ1S6DifVarnc (F = 0.54)
Benign and metastatic lymph nodes	**CZ1S6Entropy (F = 6.25), CN4S6Entropy (F = 4.98), CV2S6Entropy (F = 4.91),** **CH2S6Entropy (F = 4.9), CH3S6Entropy (F = 4.88), CZ2S6Entropy (F = 4.88),** **CN5S6Entropy (F = 4.88), CN3S6Entropy (F = 4.78), CN2S6Entropy (F = 4.75),** **CV4S6Entropy (F = 4.68)**

F (value of Fisher coefficient), InvDfMom (inverse difference moment), Teta4 (parameter θ4), Teta3 (parameter θ3), Contrast (contrast), Correlat (correlation), DifVarnc (difference variance), Entropy (entropy), RLNonUni (run length nonuniformity), GLevNonU (grey level nonuniformity).

**Table 2:** The median values of the best-suited parameters for distinguishing metastatic and non-metastatic lymph nodes and the univariate analysis results. Between the brackets, values corresponding to the interquartile range.

Entropy Variation	Non-Metastatic Lymph Nodes	Metastatic Lymph Nodes	Univariate Analysis (*p*)
CZ1S6	2.46 (2.21–2.63)	2.71 (2.41–2.9)	<0.0001
CN4S6	2.37 (2.23–2.53)	2.76 (2.67–2.92)	<0.0001
CV2S6	2.5 (2.39–2.57)	2.76 (2.69–2.85)	<0.0001
CH2S6	2.5 (2.38–2.57)	2.78 (2.67–2.86)	<0.0001
CH3S6	2.49 (2.37–2.6)	2.79 (2.69–2.9)	<0.0001
CZ2S6	2.51 (2.4–2.57)	2.79 (2.69–2.88)	<0.0001
CN5S6	2.3 (2.11–2.49)	2.75 (2.63–2.92)	<0.0001
CN3S6	2.43 (2.29–2.57)	2.78 (2.68–2.92)	<0.0001
CN2S6	2.49 (2.33–2.59)	2.78 (2.7–2.89)	<0.0001
CV4S6	2.47 (2.35–2.59)	2.77 (2.69–2.89)	<0.0001

**Table 3:** Receiver operating characteristic analysis results of the parameters that showed statistically significant results at the univariate analysis.

Entropy Variation	AUC	Significance Level	Youden Index	Associated Criterion	Sensitivity (%)	Specificity (%)
CZ1S6	0.947 (0.858–0.9833)	<0.0001	0.8148	>2.5913	88 (68.8–97.6)	93.48 (82.1–98.6)
CN4S6	0.953 (0.875–0.989)	<0.0001	0.8148	>2.5872	88 (68.8–97.5)	93.48 (82.1–98.6)
CV2S6	0.936 (0.851–0.980)	<0.0001	0.7783	>2.6486	80 (59.3–93.2)	97.83 (88.5–99.9)
CH2S6	0.933 (0.848–0.979)	<0.0001	0.7965	>2.6386	84 (63.9–95.5)	95.65 (85.2–99.5)
CH3S6	0.932 (0.847–0.978)	<0.0001	0.7783	>2.674	80 (59.3–93.2)	97.83 (88.5–99.9)
CZ2S6	0.931 (0.846–0.978)	<0.0001	0.7783	>2.6748	80 (59.3–93.2)	97.83 (88.5–99.9)
CN5S6	0.957 (0.881–0.991)	<0.0001	0.8148	>2.549	88 (68.8–97.5)	93.48(82.1–98.6)
CN3S6	0.941 (0.858–0.983)	<0.0001	0.7565	>2.6692	80 (59.3–93.2)	95.65 (85.2–99.5)
CN2S6	0.937 (0.853–0.981)	<0.0001	0.7699	>2.6823	79.17 (57.8–92.9)	97.83 (88.5–99.9)
CV4S6	0.940 (0.857–0.983)	<0.0001	0.7783	>2.6727	80 (59.3–93.2)	97.83 (88.5–99.9)

**Table 4:** Multivariate analysis of factors independently associated with the presence of metastatic lymph nodes.

Independent Variable	Coefficient	Standard Error	*p*	VIF
CZ1S6	4.0480	1.9353	0.0408	62.276
CN4S6	2.0173	2.7817	0.4712	339.770
CV2S6	−0.3968	2.2414	0.8601	99.692
CH2S6	−1.4639	2.5142	0.5626	136.360
CH3S6	2.7820	2.6989	0.3068	194.855
CZ2S6	−2.7008	2.3955	0.2641	136.320
CN5S6	0.4381	1.3913	0.7539	120.521
CN3S6	−2.0360	2.9980	0.4997	308.708
CN2S6	−1.9234	2.5207	0.4485	174.031
CV4S6	0.9889	1.8063	0.5861	93.673
**Sign. level**	<0.0001			
**R^2^**	0.587			
**R^2^ adjusted**	0.5170			
**M.R. Coef.**	0.7661			

*p*, significance level; VIF, variance inflation factor; R^2^, coefficient of determination; R^2^ adjusted, coefficient of determination adjusted for the number of independent variables in the regression model; Sign. level, significance level of the multivariate analysis; M.R. Coef., multiple correlation coefficient.
